# Let’s stop dumping cookstoves in local communities. It’s time to get implementation right

**DOI:** 10.1038/s41533-019-0160-8

**Published:** 2020-01-10

**Authors:** Evelyn A Brakema, Rianne Mjj van der Kleij, Debbie Vermond, Frederik A van Gemert, Bruce Kirenga, Niels H Chavannes, FRESH AIR collaborators, Pham Le An, Marilena Anastasaki, Azamat Akylbekov, Andy Barton, Antonios Bertsias, Pham Duong Uyen Binh, Job F M van Boven, Dennis Burges, Lucy Cartwright, Vasiliki E Chatzea, Liza Cragg, Tran Ngoc Dang, Ilyas Dautov, Berik Emilov, Irene Ferarrio, Ben Hedrick, Le Huynh Thi Cam Hong, Nick Hopkinson, Elvira Isaeva, Rupert Jones, Corina de Jong, Sanne van Kampen, Winceslaus Katagira, Jesper Kjærgaard, Janwillem Kocks, Le Thi Tuyet Lan, Tran Thanh Duv Linh, Christos Lionis, Kim Xuan Loan, Maamed Mademilov, Andy McEwen, Patrick Musinguzi, Rebecca Nantanda, Grace Ndeezi, Sophia Papadakis, Hilary Pinnock, Jillian Pooler, Charlotte C Poot, Maarten J Postma, Anja Poulsen, Pippa Powell, Nguyen Nhat Quynh, Susanne Reventlow, Dimitra Sifaki-Pistolla, Sally Singh, Talant Sooronbaev, Jaime Correia de Sousa, James Stout, Marianne Stubbe Østergaard, Aizhamal Tabyshova, Ioanna Tsiligianni, Tran Diep Tuan, James Tumwine, Le Thanh Van, Nguyen Nhu Vinh, Simon Walusimbi, Louise Warren, Sian Williams

**Affiliations:** 1 Department of Public Health and Primary Care, Leiden University Medical Center, Leiden, the Netherlands; 2 Department of General Practice & Elderly Care Groningen Research Institute for Asthma and COPD (GRIAC), University Medical Center Groningen, University of Groningen, Groningen, The Netherlands; 3 Unit of Global Health, Department of Health Sciences, University Medical Center Groningen, University of Groningen, Groningen, The Netherlands; 4 Department of Medicine and Makerere Lung Institute, Makerere University, Kampala, Uganda; 5 University of Medicine and Pharmacy, Ho Chi Minh, Vietnam; 6 Clinic of Social and Family Medicine, School of Medicine, University of Crete, Heraklion, Greece; 7 Ministry of Health of the Kyrgyz Republic, National Center of Cardiology and Internal Medicine, Bishkek, Kyrgyzstan; 8 Faculty of Medicine and Dentistry, University of Plymouth, Plymouth, UK; 9 University of Groningen, University Medical Center Groningen, Groningen, The Netherlands; 10 Department of Pediatrics, University of Washington School of Medicine, Seattle, WA USA; 11 International Primary Care Respiratory Group, London, UK; 12 European Lung Foundation, Sheffield, UK; 13 Imperial College London, London, UK; 14 The Research Unit for General Practice and Section of General Practice, Department of Public Health, Copenhagen University, Copenhagen, Denmark; 15 Global Health Unit, The Department of Paediatrics and Adolescent Health, Juliane Marie Center, Copenhagen University Hospital “Rigshospitalet”, Copenhagen, Denmark; 16 National Centre for Smoking Cessation and Training, Dorchester, UK; 17 Usher Institute of Population Health Sciences and Informatics, University of Edinburgh, Edinburgh, UK; 18 Coventry University, Coventry, UK; 19 School of Medicine, University of Minho, Braga, Portugal

**Keywords:** Preventive medicine, Chronic obstructive pulmonary disease, Disease prevention, Health policy, Public health

We most welcome the comment by Thakur, van Schayck and Boudewijns^1^ on our article on the effects and acceptability of implementing improved cookstoves.^2^ Adoption rates of improved cookstoves by local communities are often strikingly low. The authors underline the urge to advance cookstove implementation strategies, and reinforce the approach used in the FRESH AIR project.^2^ They highlight several important factors to increase adoption success and call for further research on the topic. We want to build on this comment by reflecting on decades of substantial discrepancies between the disappointing adoption rates of improved cookstoves, and the subsequent failure to adapt implementation strategies accordingly. We argue that it is not necessarily the lack of evidence that impedes the success of implementation strategies for improved cookstoves. Moreover, it is the lack of use of the evidence by implementors. We propose several ideas for overcoming this evidence-to-practice gap.

## The need for improved cookstoves

Improved cookstoves have been on the market for over seven decades. The rationale for their need is simple: three billion people worldwide rely on solid fuels (e.g., wood and coal) as their main energy source.^3^ Burning solid fuels in open fires or inefficient stoves has detrimental health and environmental consequences. Inhalation of polluted air is ranked the fifth risk of deaths and sixth risk for disability-adjusted life-years globally,^4^ as it causes among others impaired lung development, respiratory infections and cardiovascular disease.^5–7^ Besides, solid fuel use causes widescale deforestation and up to 25% of global black carbon emissions; black carbon emissions are the largest contributors to climate change after carbon dioxide emissions.^8,9^ Hence, developing a technical solution to reduce air pollution and fuel consumption and distributing it among local communities should solve the problem. Right?

## The discrepancy between implementation evidence and implementation strategies

Improved stoves, with their higher combustion efficiency, would generate less smoke and consume less fuel. Therefore, improved stoves as a solution to the problems above seems as plausible to reasonable minds as it seems appealing to idealists’ emotions (and idealism drives many researchers to do what they do, after all). As Aristotle knew already, this combination of logos and pathos is a powerful persuader, which could explain the numerous attempts to push cookstoves into local markets despite the accumulating evidence that their adoption is failing.^7,10^ Improved cookstoves—outside of the laboratory setting—have hardly demonstrated any consistent improvements in health outcomes (high-quality articles reported no health benefits, some health benefits, or inconclusiveness).^10–14^ In the real world, clean cookstoves have turned out to be incredibly challenging to implement. Adoption rates frequently remain unreported, but studies that report on adoption success use descriptions as ‘largely discouraging’, ‘a mere 10%’, ‘only 4%’, ‘rare’, and ‘very low’.^15–19^ If adopted, improved stoves are often used concurrently with traditional stoves (known as stove-stacking), which may lead to even higher levels of air pollution and fuel consumption.^20^ Although these observations and analyses of implementation factors were already described in the eighties and nineties,^19,21–24^ implementation strategies and adoption rates generally appear not to have changed accordingly.

## How to move forward in implementation?

Facing the facts: the adoption of improved cookstoves by local communities has largely failed since the stoves appeared on the market 70 years ago, draining funds available for resource-limited settings. Meanwhile, the health and environmental problems related to solid fuel use have become more urgent than ever.^25,26^ Community-focused approaches, creation of public awareness on the risks of kitchen smoke, provision of stove usage information, assurance of maintenance, involvement of women and an appropriate business model were outlined as implementation facilitators by Thakur et al.^1^ Other consistently reported, related, factors are characteristics of the stove (e.g., costs or real-world effectiveness), compatibility between the stove and local needs and perceptions (e.g., meeting taste preferences to avoid stove-stacking), and favourable policies (e.g., laws, regulations, and subsidies), as outlined in existing reviews into barriers and facilitators to the adoption of improved cookstoves.^10,20,27–30^ (These reviews referred to were among the most recent ones; however, we are aware of over 20 existing cookstove implementation reviews since 2010). Interestingly, these factors do not differ from the factors described in reviews >30 years ago.^19,21–24^ We agree with Thakur et al. that generating new evidence on implementation is useful, but only provided that implementation strategies and processes are reported in detail, adoption rates and stove-stacking are systematically and objectively assessed,^31^ and follow-up time is 4 years or more, as underlined by recent Nobel Prize winner Esther Duflo and her colleagues.^11^ Although this can be challenging (in FRESH AIR our funding was only adequate for six to twelve months of follow-up), this should be the norm for future implementation studies.

However, above all, this comment is a call to actually use the existing evidence in the design and execution of implementation strategies for improved stoves. Doing so requires efforts from all stakeholders involved. To facilitate designs of effective implementation strategies, the existing bulge of cookstove implementation evidence should be consolidated in an easy-to-use way, such as a state-of-the-art implementation tool. The tool should then be applied in future cookstove implementation projects and researchers should ensure to constantly update it according to the latest evidence and priorities.^32^ Researchers should also connect to brokers in large network organisations, such as the Clean Cooking Implementation Science Network, the Clean Cooking Alliance (formerly Global Alliance for Clean Cookstoves) and the World Health Organization (WHO). These organisations should promote and distribute the implementation tool to make it well-known and easily available. Policymakers should ensure to consult it for decision-making. Furthermore, funders, non-governmental organisations, and development institutions such as the World Bank should exclusively grant support for proposals and project plans with adequate implementation strategies that address the implementation factors in the tool. Lastly, carbon credit (offset) projects should incentivise on improved cookstove adoption instead of distribution. Collaborative efforts and constant networking for knowledge exchange between all stakeholders are vital, to ensure everyone is on the same, up-to-date, page. As a start, we have reached out to Thakur, van Schayck and Boudewijns to team up and start developing this implementation tool.

The steps above could facilitate idealism to team up with evidence-based realism and help to get implementation right. Only then we can actually assess whether improved stoves are consistently effective in the real world, acknowledging that challenges persist even with perfectly implemented improved cookstoves (like decreased levels of household air pollution that remain above the WHO recommended levels^10^). However, until clean fuels such as electricity are affordable and available for everyone (or until long-term research into well-implemented stoves proves us differently), we should strive for improved, evidence-based implementation of improved cookstoves, to ultimately improve environmental and health outcomes.
